# Antidepressant Effects of Ginsenoside Rc on L-Alpha-Aminoadipic Acid-Induced Astrocytic Ablation and Neuroinflammation in Mice

**DOI:** 10.3390/ijms25179673

**Published:** 2024-09-06

**Authors:** Dohyung Kwon, Yunna Kim, Seung-Hun Cho

**Affiliations:** 1 Department of Clinical Korean Medicine, Graduate School, Kyung Hee University, Seoul 02447, Republic of Korea; curtail92@gmail.com; 2 College of Korean Medicine, Kyung Hee University, Seoul 02447, Republic of Korea; yunna.anna.kim@khu.ac.kr; 3 Department of Neuropsychiatry of Korean Medicine, Kyung Hee University Medical Center, Kyung Hee University, Seoul 02447, Republic of Korea; 4 Research Group of Neuroscience, East-West Medical Research Institute, WHO Collaborating Center, Kyung Hee University, Seoul 02447, Republic of Korea

**Keywords:** astrocyte, depression, ginsenoside Rc, L-alpha-aminoadipic acid, neuroinflammation

## Abstract

Depression is a prevalent and debilitating mental disorder that affects millions worldwide. Current treatments, such as antidepressants targeting the serotonergic system, have limitations, including delayed onset of action and high rates of treatment resistance, necessitating novel therapeutic strategies. Ginsenoside Rc (G-Rc) has shown potential anti-inflammatory and neuroprotective effects, but its antidepressant properties remain unexplored. This study investigated the antidepressant effects of G-Rc in an L-alpha-aminoadipic acid (L-AAA)-induced mouse model of depression, which mimics the astrocytic pathology and neuroinflammation observed in major depressive disorder. Mice were administered G-Rc, vehicle, or imipramine orally after L-AAA injection into the prefrontal cortex. G-Rc significantly reduced the immobility time in forced swimming and tail suspension tests compared to vehicle treatment, with more pronounced effects than imipramine. It also attenuated the expression of pro-inflammatory cytokines (TNF-α, IL-6, TGF-β, lipocalin-2) and alleviated astrocytic degeneration, as indicated by increased GFAP and decreased IBA-1 levels. Additionally, G-Rc modulated apoptosis-related proteins, decreasing caspase-3 and increasing Bcl-2 levels compared to the L-AAA-treated group. These findings suggest that G-Rc exerts antidepressant effects by regulating neuroinflammation, astrocyte–microglia crosstalk, and apoptotic pathways in the prefrontal cortex, highlighting its potential as a novel therapeutic agent for depression.

## 1. Introduction

Depression is a highly prevalent mental disorder, with a lifetime prevalence of 10.8% worldwide [1], and causes psychological and physical symptoms [2]. Antidepressants are among the most commonly prescribed drugs in the United States, with sales surpassing several billion USD annually, and 11% of American adults consume them [3]. However, new therapeutic strategies remain in demand because current treatments are limited and approximately 30% of patients with depression are treatment-resistant [4]. Recently, an umbrella systematic review revealed that the association between depression and serotonin, a major target of current first-line medications, was not concrete [5].

Previous studies on the brains of patients with major depressive disorder (MDD) have revealed a decreased density of astrocytes, while neurons simply atrophied or did not change significantly [6,7,8]. This astrocytic abnormality can cause depression by reducing the uptake and circulation of glutamate, the major excitatory amino acid neurotransmitter that maintains a balance with gamma-aminobutyric acid, leading to its accumulation [9]. A recent study reported that astrocyte ablation in mice increased the number of microglia with ramified branches and the levels of tumor necrosis factor-alpha (TNF-α) and interleukin (IL)-6 but decreased the level of IL-10, which is indicative of neuroinflammation and was accompanied by depression-like behaviors. This alteration in the microglia was driven by astrocyte-derived transforming growth factor-beta (TGF-β), and in the absence of TGF-β there was no change in the formation of microglial branches or the microglia-mediated inflammatory response [10].

Several studies have reported on the contribution of neuroinflammation to the pathology of depression. Many inflammatory markers, such as IL-6 and TNF-α, exhibited increased levels in the hippocampus and prefrontal cortex (PFC) in major models of depression, such as chronic social defeat stress, chronic restraint stress, and chronic unpredictable mild stress [11]. As the most abundant glial cells in the brain, astrocytes are deeply involved in the inflammatory pathology of depression and affect various inflammatory cytokines [12]. Lipocalin-2 (LCN2), a pro-inflammatory cytokine mediator, has been associated with depression and is often accompanied by elevated levels of IL-6 and TNF-α [13,14]. LCN2 derived from astrocytes has been implicated in the development of neuroinflammation [15]. LCN2 has been found to be upregulated in both human patients with depression and animal models of depression induced by stress [14,16,17,18,19]. Therefore, medications that regulate astrocytic pathology and neuroinflammatory markers, including LCN2, IL-6, and TNF-α, are promising for alleviating depressive symptoms.

*Panax ginseng* Meyer (*P. ginseng*) is a herbal plant that is used to treat various diseases, including neuropsychiatric disorders such as MDD. Ginsenosides are the principal active ingredients of *P. ginseng* and are unique to the ginseng species, and extensive studies have revealed the antidepressant effects of *P. ginseng* extract [20,21,22,23]. Among the components of *P. ginseng*, ginsenoside Rb1 [23], ginsenoside Rb3 [24], ginsenoside Rf [25], ginsenoside Rg1 [26,27], compound K [23], and 20 (S)-protopanaxadiol [28] have been reported to improve depression-like behavior in rodents.

Over 50 types of ginsenosides are known, and ginsenoside Rc (G-Rc) is one of the major ginsenosides, comprising 90% of all ginsenosides, including Rb1, Rb2, Rd, Re, and Rg1 [29]. G-Rc is classified as a protopanaxadiol and exhibits anti-inflammatory [30,31,32,33], antioxidative [32,34], and protective effects on the central nervous system [35]. These results suggest that G-Rc can be used to treat neurological disorders. However, to the best of our knowledge, no studies have investigated the effects of G-Rc on MDD. Therefore, in this study, an L-alpha-aminoadipic acid (L-AAA)-induced mouse model was used to mimic the astrocytic pathology of depression and neuroinflammation, and to observe whether G-Rc treatment significantly ameliorated depression-like behavior and histological changes in this model.

## 2. Results

### 2.1. Effects of G-Rc Administration on the Depression-like Behavior of L-AAA-Induced Depression in Mice

Based on the evidence that L-AAA infusion into the PFC induces depression-like behavior and histological changes similar to those observed in patients with MDD, such as transient astrocytic ablation and local glial degeneration, we employed the L-AAA-induced depression model in this study to investigate the antidepressant effects of G-Rc [9,36,37].

**Figure 1:**
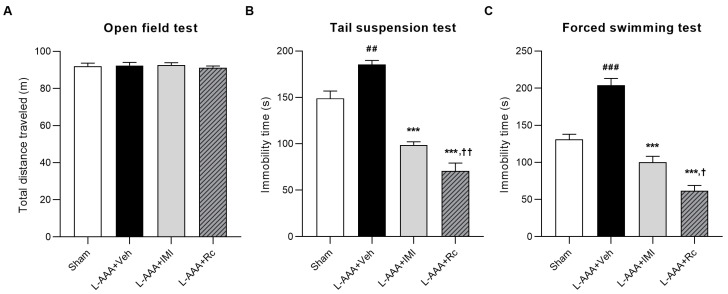
Assessment of locomotor activity and depression-like behavior: (**A**) Locomotion of mice was evaluated using an open-field test. The effects of G-Rc on depressive behavior were assessed using the (**B**) tail suspension test and (**C**) forced swimming test. Immobility time in both tests implies depression-like behavior. Results are expressed as the mean ± SEM; N = 5 per group; ## *p* < 0.01 and ### *p* < 0.001, significantly different from the sham control group (sham); *** *p* < 0.001, significantly different from the vehicle-treated group (L-AAA + Veh); † *p* < 0.05 and †† *p* < 0.01, significantly different from the IMI-treated group (L-AAA + IMI). G-Rc, ginsenoside Rc; L-AAA, L-alpha-aminoadipic acid; TST, tail suspension test; FST, forced swimming test; SEM, standard error of the mean; IMI, imipramine.

The open-field test (OFT) was used to assess potential locomotor impairments caused by the surgical procedure. No significant differences were observed between groups (*p* > 0.05; Figure 1A). This suggests that intracerebral infusion did not damage sensorimotor function.

To assess the effects of G-Rc on the depression-like behavior of L-AAA-infused mice, the forced swimming test (FST) and tail suspension test (TST) were performed. In both tests, the immobility time of vehicle-treated mice was significantly increased compared with that of the sham control (sham) group (*p* < 0.01), whereas the IMI-treated group showed a decrease in immobility time compared with that of the vehicle-treated group (TST, *p* < 0.01; FST, *p* < 0.001). The administration of G-Rc significantly decreased the immobility time (*p* < 0.001), which was greater than that in the imipramine (IMI)-treated group (TST, *p* < 0.01; FST, *p* < 0.05). These data suggest that G-Rc can reduce depressive behavior induced by L-AAA infusion (Figure 1B,C).

### 2.2. Anti-Inflammatory Effects of G-Rc on L-AAA-Infused Mice

**Figure 2:**
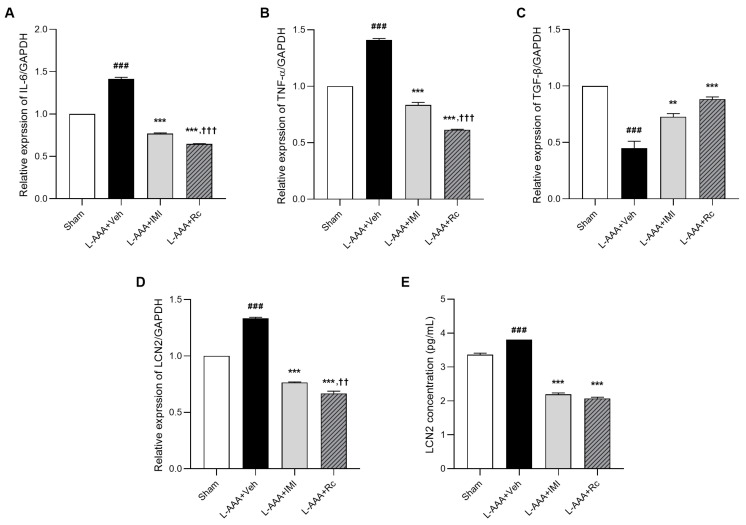
The anti-inflammatory effects of G-Rc were assessed using real-time PCR and ELISA: (**A**) Real-time PCR results for TNF-α, (**B**) IL-6, (**C**) TGF-β, and (**D**) LCN2 are presented. (**E**) ELISA results for LCN2 are also shown. Results are expressed as the mean ± SEM; N = 3 per group; ### *p* < 0.001, significantly different from the sham control group (sham); ** *p* < 0.01 and *** *p* < 0.001, significantly different from the vehicle-treated group (L-AAA + Veh); †† *p* < 0.01 and ††† *p* < 0.001, significantly different from L-AAA + IMI. G-Rc, ginsenoside Rc; L-AAA, L-alpha-aminoadipic acid; SEM, standard error of the mean; ELISA, enzyme-linked immunosorbent assay; TGF-β, transforming growth factor-beta; TNF-α, tumor necrosis factor-α; IL-6, interleukin-6; LCN2, lipocalin-2; IMI, imipramine.

To assess the anti-inflammatory effects of G-Rc, the mRNA expression of IL-6, TNF-α, TGF-β, and LCN2 was measured using real-time PCR. The mRNA expression of IL-6 and TNF-α, the most relevant pro-inflammatory cytokines with LCN2, increased significantly in mice treated with the vehicle (*p* < 0.001), whereas the increases in these levels were inhibited with the G-Rc and IMI treatments (*p* < 0.001). G-Rc treatment significantly reduced inflammatory responses to a greater extent than IMI treatment (*p* < 0.001; Figure 2A,B).

In a previous study, it was demonstrated that TGF-β, secreted by astrocytes, regulates microglial ramification and the expression of pro-inflammatory cytokines, including TNF-α [10]. Thus, the change in TGF-β mRNA expression was quantified using real-time PCR. The mRNA expression of TGF-β was found to significantly diminish following L-AAA injection (*p* < 0.001). However, treatment with G-Rc and IMI resulted in a significant increase in the expression level of TGF-β (L-AAA + IMI, *p* < 0.01; L-AAA + Rc, *p* < 0.001, Figure 2C).

The level of LCN2, a pro-inflammatory cytokine mediator known to be associated with depression [38,39], was increased in vehicle-treated mice as assessed using real-time PCR (*p* < 0.001). Similarly, the G-Rc- and IMI-treated groups showed suppressed LCN2 expression compared with that in the vehicle-treated group (L-AAA + IMI, *p* < 0.001; L-AAA + Rc, *p* < 0.001), and G-Rc was more effective than IMI (*p* < 0.01, Figure 2D). To quantify the protein expression of LCN2, enzyme-linked immunosorbent assay (ELISA) was also used (Figure 2E). This result was similar to that of real-time PCR; the level of LCN2 increased in the vehicle-treated group (*p* < 0.001) and decreased in the G-Rc- and IMI-treated groups (*p* < 0.001). These results provide evidence that G-Rc reduces inflammatory responses via LCN2 regulation in astrocyte-ablated mouse models.

### 2.3. Effects of G-Rc on Downregulation of GFAP and Upregulation of IBA-1 Caused by L-AAA Infusion

**Figure 3:**
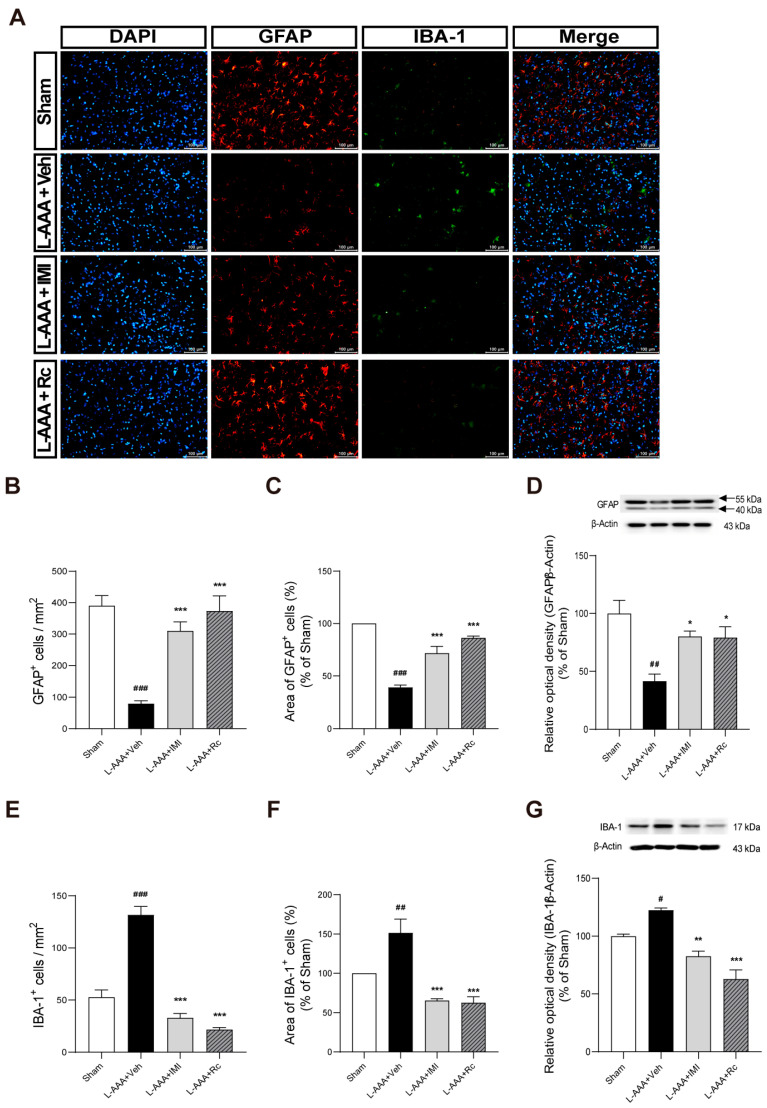
Effects of G-Rc on astrocyte and microglial markers following L-AAA administration: (**A**) The suppressive effects of G-Rc on L-AAA administration were evaluated via immunofluorescence staining. Representative results from immunohistochemistry are presented showing GFAP (red), IBA-1 (green), and DAPI (blue). After L-AAA injection, (**B**,**C**) the number and the area of GFAP-positive astrocytes (red) decreased, whereas (**E**,**F**) the number and the area of IBA-1-positive microglial cells (green) increased. However, the histopathological deficits were reduced in the G-Rc-treated (L-AAA + Rc) and IMI-treated (L-AAA + IMI) groups. Western blot analyses of (**D**) GFAP and (**G**) IBA-1 protein levels, respectively, were performed, and the results were consistent with the immunofluorescence findings, confirming the effects of L-AAA and G-Rc treatment on GFAP and IBA-1 expression. Scale bars are set at 100 μm. Quantitative data are expressed as the mean ± SEM; N = 3 per group; # *p* < 0.05, ## *p* < 0.01, ### *p* < 0.001, significantly different from the sham control group (sham); * *p* < 0.05, ** *p* < 0.01, *** *p* < 0.001, significantly different from the vehicle-treated group (L-AAA + Veh); G-Rc, ginsenoside Rc; L-AAA, L-alpha-aminoadipic acid; GFAP, glial fibrillary acidic protein; IBA-1, ionized calcium-binding adaptor molecule 1; IMI, imipramine.

After L-AAA injection, decreased levels of glial fibrillary acidic protein (GFAP) and increased levels of adjacent ionized calcium-binding adapter molecule 1 (IBA-1) were observed using immunofluorescence staining (Figure 3A). The number and the area of GFAP-positive cells significantly decreased after L-AAA injection (*p* < 0.001, Figure 3B,C), while the number and the area of IBA-1-positive cells significantly increased compared to the sham group (*p* < 0.001, Figure 3E,F). Conversely, mice treated with G-Rc and IMI displayed increased GFAP levels and decreased IBA-1 levels (*p* < 0.001). These results indicate that G-Rc alleviates astroglial impairment, potentially through the modulation of astrocyte–microglia crosstalk.

To confirm the quantitative changes in the expression of GFAP and IBA-1, Western blot analysis was performed. The results showed a significant decrease in GFAP protein levels in the L-AAA + Veh group compared to the sham group (*p* < 0.01). G-Rc treatment significantly increased GFAP levels compared to the L-AAA + Veh group (*p* < 0.05), supporting the immunofluorescence observations of astrocyte recovery (Figure 3D). Additionally, the Western blot results demonstrated a significant increase in IBA-1 protein levels in the L-AAA + Veh group compared to the sham group (*p* < 0.05). G-Rc treatment significantly reduced IBA-1 levels compared to the L-AAA + Veh group (*p* < 0.001), confirming the immunofluorescence findings of reduced microglial activation (Figure 3G).

Western blot analysis of neuronal nuclei antigen (NeuN) protein levels showed no significant differences between the sham, L-AAA-treated, and G-Rc-treated groups (*p* > 0.05; Supplementary Figure S1). This result indicates that neither L-AAA administration nor subsequent G-Rc treatment significantly altered the overall neuronal population in the prefrontal cortex, consistent with previous findings in both L-AAA models and clinical depression studies [7,25,36,40].

### 2.4. Effects of G-Rc on Apoptosis-Related Proteins Following L-AAA Infusion

**Figure 4:**
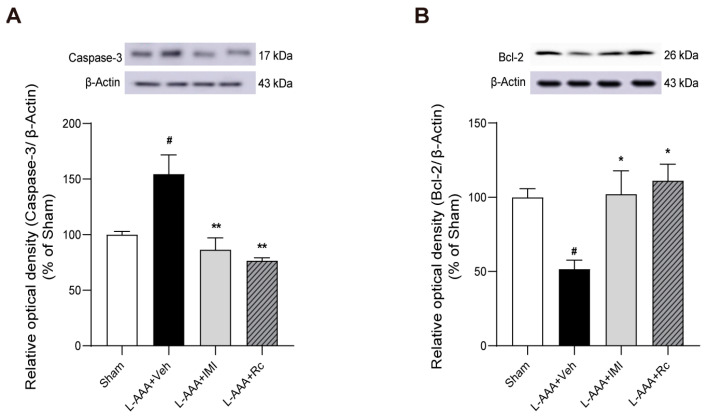
Suppressive effects of G-Rc on apoptosis-related proteins following L-AAA administration: Western blot analysis of (**A**) caspase-3 protein levels and (**B**) Bcl-2 protein levels. Quantitative data are expressed as the mean ± SEM; N = 3 per group; # *p* < 0.05, significantly different from the sham control group (sham); * *p* < 0.05, ** *p* < 0.01, significantly different from the vehicle-treated group (L-AAA + Veh). G-Rc, ginsenoside Rc; L-AAA, L-alpha-aminoadipic acid; IMI, imipramine.

Given the potential role of apoptosis in depression and neuroinflammation, the effects of G-Rc on key apoptosis-related proteins were examined. To investigate this, Western blot analyses of caspase-3 and Bcl-2 were performed. The results showed a significant increase in caspase-3 levels in the L-AAA + Veh group compared to the sham group (*p* < 0.05). G-Rc treatment significantly decreased caspase-3 levels compared to the L-AAA + Veh group (*p* < 0.01) (Figure 4A). Bcl-2 levels were significantly decreased in the L-AAA + Veh group compared to the sham group (*p* < 0.05). G-Rc treatment significantly increased Bcl-2 levels compared to the L-AAA + Veh group (*p* < 0.05) (Figure 4B). These findings suggest that G-Rc may modulate apoptotic pathways in L-AAA-induced depression, potentially contributing to its therapeutic effects.

## 3. Discussion

To explore the antidepressant effects of G-Rc, we assessed its effects on neurobehavioral changes and observed histopathological alterations in the PFC of mice. Animal experiments were conducted to observe the effects of G-Rc on depression-like behavior in the L-AAA-induced mouse model, and G-Rc treatment significantly improved the relevant symptoms, as shown in behavioral tests. The G-Rc-treated group exhibited anti-inflammatory effects by reducing the levels of inflammatory response biomarkers and alleviating astrocytic degeneration. Furthermore, G-Rc demonstrated anti-apoptotic effects, as evidenced by decreased levels of the pro-apoptotic protein caspase-3 and increased levels of the anti-apoptotic protein Bcl-2. Thus, G-Rc effectively ameliorates depression by regulating neuroinflammation, astrocytes, and apoptotic pathways.

To the best of our knowledge, G-Rc has not been investigated in animal models of depression. Previous studies on other ginsenosides mostly used chronic stress models. Ginsenosides Rb1, Rb3, Rd, Rg2, Rg3 [24,41,42,43], and Rg1 [27,44,45,46,47] have been reported to improve depression-like behavior in a chronic unpredictable mild stress model. Furthermore, ginsenoside Re in the chronic immobilization stress model [48], ginsenosides Rg3 and Rg5 in the chronic social defeat stress model [49,50], and majonoside-R1 and majonoside-R2 in a socially isolated depression mouse model [51] alleviated depression-like behaviors. A few studies used inflammation-induced models of depression, such as ginsenosides Rg1 and Rh2 in lipopolysaccharide-challenged mouse models [26,52]. Our findings contribute to the evidence of the antidepressant effect of G-Rc.

Infusion of L-AAA into the PFC causes depression-like behavior and induces histological features in the brains of patients with MDD, such as transitory ablation of astrocytes, which causes local glial degeneration [9,36,37]. A reduction in the density and area of GFAP-positive astrocytes was observed in the ventral prefrontal white matter of both humans with MDD and mice subjected to chronic unpredictable stress [53]. Postmortem studies consistently report a loss in astrocyte density rather than neurons in patients with MDD [7,40]. Decreased densities of GFAP or vimentin-immunoreactive astrocytes have been observed in the prefrontal cortex, hippocampus, caudate nucleus, and amygdala in postmortem studies of MDD [53,54,55,56]. Similarly, studies using Golgi staining and cresyl violet staining have shown a general reduction in glial density throughout the brain in patients with MDD [57,58]. With regard to the function of astrocytes, postmortem data indicate that astrocytes in MDD exhibit reduced interactions with other cell types, as demonstrated by lower expression of glutamate transporters, neurotrophic factors, and gap junctions [40,59].

Considering these aspects, a reduction in the number and altered function of astrocytes would be more crucial characteristics of MDD, compared to morphology. Astrocyte density and dysfunction in diverse regions of the brain are observed in patients with MDD, and this L-AAA-infused model may be a reasonable alternative to animal models of MDD [54,55,60]. L-AAA is an analogue of glutamate that selectively ablates astrocytes by entering the cells via sodium-dependent excitatory amino acid transporters and cysteine/glutamate antiporters [61]. Upon entering astrocytes, L-AAA inhibits glutamine synthetase and gamma-glutamylcysteine synthetase, thereby disrupting glutamate cycling [37]. As a gliotoxin, it specifically ablates astrocytes, leading to their degeneration without affecting neurons. Studies have demonstrated that the infusion of L-AAA into the PFC of rodents induces behaviors associated with depression, such as anhedonia, anxiety, and helplessness [62]. A previous study has suggested that the L-AAA-infused model has the advantage of evaluating the effects of the intervention by equally modulating changes in respective animal subjects [25]. While astrocytes can be detected by various markers, such as GFAP, vimentin, AlDh1L1, and S100-β, GFAP was considered to be an appropriate marker for this study, as most studies on the changes in astrocytes in postmortem MDD patients have used GFAP [40]. Based on these findings, we adopted the L-AAA-induced astrocyte ablation model and used GFAP to assess the change in the injected region following drug administration.

The TST and FST are widely used to assess depressive behavior in animals, such as mice, and are sensitive to the administration of antidepressant drugs [63,64]. The immobility time during these behavioral tests indicates depression-like behavior [65], and in this study astrocyte ablation in the L-AAA-infused mice showed an increased duration of immobility. The significant reduction in immobility time observed in the L-AAA + Rc group compared to the sham control in both the TST and FST suggests that G-Rc may have potent antidepressant effects, beyond merely reversing L-AAA-induced depressive-like behavior. In the TST, this effect was also seen with imipramine treatment, while in the FST, only G-Rc showed this significant reduction. As no differences were observed in the open-field test, these results likely reflect mood-specific effects rather than general changes in locomotor activity.

While decreasing depression-like behaviors in animals under L-AAA infusion, G-Rc decreased pro-inflammatory cytokines such as IL-6 and TNF-α. A meta-analysis on a total sample of 10,249 individuals revealed that the mean levels of seven inflammatory markers, including IL-6 and TNF-α, were elevated in patients with depression in comparison to healthy controls [66]. The administration of antidepressant medication was found to result in a notable reduction in the peripheral levels of IL-6, TNF-α, IL-10, and CCL-2 [67]. Studies have reported that inflammatory markers are upregulated in several animal models of MDD [11]. Furthermore, astrocyte loss also increased the expression of IL-6 and TNF-α [68,69]. Therefore, the expression of these pro-inflammatory cytokines was evaluated in order to assess neuroinflammation with regard to depression. After L-AAA injection, IL-6 and TNF-α levels increased, whereas G-Rc administration suppressed their elevation.

LCN2, also known as neutrophil gelatinase-associated lipocalin, is an acute-phase protein involved in inflammation that plays a central role as a major mediator of inflammation and is an indicator of depression [17,70,71]. Consequently, we demonstrated that the increase in LCN2 expression was attenuated in the G-Rc group. Previous studies have shown that LCN2 secreted from astrocytes resulted in the degradation of neurons [72,73]. LCN2 expression is increased in the ovariectomized rat forced swim stress- and repeated social defeat stress-induced mouse models of depression [18,19,38]. Recently, LCN2 was suggested as a marker of late-life depression [17,74,75]. LCN2, a mediator of inflammation, is associated with depressive symptoms associated with somatic disorders [70,71,76,77]. LCN2 is regulated by astrocytes, which can be a profound factor in depression owing to decreased astrocytes in the brain [78,79]. LCN2 significantly upregulated the expression of the pro-inflammatory cytokines IL-6 and TNF-α, particularly in response to stimuli such as lipopolysaccharide [80,81]. In Lcn2-deficient macrophages, there was a significant decrease in the levels of IL-6 and TNF-α [82]. This upregulation involved the activation of various pathways, such as NF-κB and mitogen-activated protein kinase [83]. Elevated levels of LCN2, TNF-α, and IL-6 were linked to various CNS pathologies, indicating that LCN2 may represent a potential target for therapeutic strategies to mitigate neuroinflammation [81].

Astrocytes are the most abundant cell type in the brain, and they protect and support neurons and regulate microglial activation and ramification [84]. Astrocytes inhibit microglia-mediated inflammatory responses through the secretion of TGF-β, and astrocyte ablation causes depression-like behavior [10]. The TGF-β pathway includes numerous genes that play critical roles in cell growth, differentiation, migration, and apoptosis [85]. Given the diverse functions of TGF-β in the nervous system, including its role in neuroplasticity and neuroprotection, it is evident that TGF-β signaling is also involved in the development of depression [86]. Patients suffering from depression had low expression of the TGF-β1 genotype [87]. In mice with chronic unpredictable mild stress, immobility time and sucrose preference were correlated with levels of IL-6 and TGF-β [88]. Antidepressants such as tianeptine and venlafaxine significantly increased TGF-β and its receptors in the frontal cortex and hippocampus of prenatally stressed rats [89]. TGF-β1 was decreased in the Lcn2-positive cell lines, but not in Lcn2-negative cell lines, suggesting that LCN2 engages in downstream TGF-β1 signaling [90]. A previous study has shown that astrocyte-derived TGF-β promoted the formation of microglial sub-branches and protuberances and regulated the cell body size [10]. In this study, anti-inflammatory effects were assessed based on the degree of astrocyte recovery, and G-Rc treatment significantly reduced astroglial impairment. This suggests that G-Rc influences a wide range of inflammatory changes, from astrocytes to microglia, as astrocyte–microglia crosstalk is important in neurological pathologies. Even though this study did not examine the co-localization of TGF-β with either astrocytes or microglia, it is plausible that TGF-β serves as a link between astrocyte loss and the accompanied microglial activation.

To investigate the potential effects of G-Rc on apoptotic pathways, we examined the expression of caspase-3 and Bcl-2. Our results showed that L-AAA injection significantly increased caspase-3 levels and decreased Bcl-2 levels, indicating an upregulation of pro-apoptotic signaling. This finding is consistent with previous studies demonstrating that L-AAA induces apoptosis in astrocytes [91,92]. G-Rc treatment significantly decreased caspase-3 levels and increased Bcl-2 levels compared with the L-AAA-treated group, suggesting a protective effect against apoptosis. This anti-apoptotic effect of G-Rc may be related to its modulatory effects on neuroinflammation and astrocyte function, as shown by decreased levels of LCN2, an inflammatory mediator that is known to induce apoptosis by upregulating pro-apoptotic proteins such as Bcl-2-interacting mediator (BIM) of cell death protein [73,93]. In the absence of survival cytokines, the induction of LCN2 is accompanied by increases in pro-apoptotic Bcl-2 family members such as Bax and BIM, which contribute to mitochondrial membrane permeabilization and subsequent apoptosis [94,95,96]. By promoting cell survival through increased Bcl-2 expression and decreased caspase-3 activation, G-Rc may help maintain astrocytes’ integrity and function, potentially contributing to its antidepressant effects.

G-Rc, a bioactive compound in *P. ginseng*, exhibits a broad range of physiological effects, including on inflammation [30,31,32,33], oxidative stress [32,34], diabetes [97,98], obesity [99], GABA receptor activity [100], myocardial protection [30,31,32,101], and sperm motility [102]. Despite extensive research, investigations into the neurological effects of G-Rc have been limited. G-Rc has demonstrated anti-inflammatory effects in animal models of myocardial injury and arthritis, gastritis, and hepatitis by inhibiting pro-inflammatory cytokines and modulating signaling pathways [30,31,32,33]. G-Rc suppressed the expression of TNF-α, IL-6, and IL-1β in myocardial injury models via TANK-binding kinase 1/IκB kinase ε/interferon regulatory factor-3 and p38/ATF-2 signaling, as well as Nrf2/HO-1 signaling [30,31,32]. It also reduced inflammatory symptoms in mouse models of arthritis, gastritis, and hepatitis by lowering TNF-α and IL-6 production while increasing IL-10 [33]. Although its anti-inflammatory effects in the nervous system have not been reported, G-Rc modulated GABA(A) receptor channels in cell lines, enhancing GABA-induced currents, suggesting its potential effect on neurological functions [100]. Given its effect on GABA, further investigation of its effects on other neurotransmitters is warranted.

In this study, a G-Rc dosage of 20 mg/kg was chosen based on research on the effects of other ginsenosides on the nervous system, as no previous studies have specifically examined the effects of G-Rc on central nervous system disorders in animals [20,103,104,105,106]. Notably, studies on the antidepressant effects of various ginsenosides have typically used dosages ranging from 10 to 50 mg/kg, with 20 mg/kg being the most common dosage.

This study had some limitations. We focused on specific aspects of depression, and other mechanisms of action of G-Rc should be evaluated to determine its antidepressant effects. To mitigate the potential limitations associated with the small sample size in the respective analyses, multiple analytical approaches were employed. Specifically, both immunofluorescence staining and Western blot analyses were conducted to quantify GFAP and IBA-1 protein expression, thereby enhancing the robustness of the findings. Although *P. ginseng* and most ginsenosides have been proven to be safe [107], further research on their toxicity should be conducted before G-Rc can be used as a novel antidepressant. While this study examined apoptosis-related proteins as indicators of potential cytotoxicity, direct cytotoxicity assays were not performed. Future studies should include comprehensive cytotoxicity evaluations of G-Rc to fully assess its safety profile and potential for clinical application.

## 4. Materials and Methods

### 4.1. Animals

Eight-week-old male C57BI/6 mice (Young Bio, Inc., Seongnam, Republic of Korea) weighing 20–22 g were used. The mice were housed in acrylic cages (26 × 42 × 18 cm) with controlled temperature (22 ± 2 °C) and relative humidity (60 ± 10%). The mice were given free access to water and food and were constantly monitored under a 12 h light/dark cycle. All behavioral tests were conducted between 9:00 and 17:00. All experiments were approved by Kyung Hee University Medical Center Institutional Animal Care (approval number: KHMC-IACUD 2019-030).

### 4.2. Drug Administration

**Figure 5:**
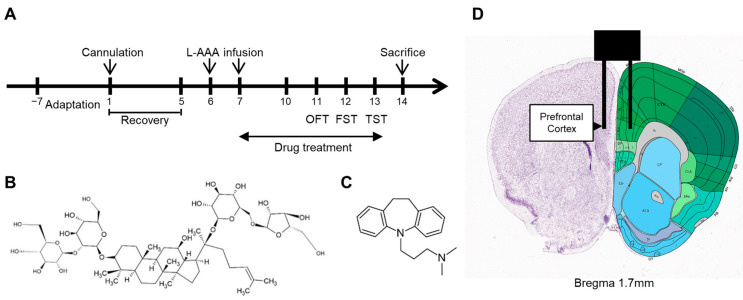
(**A**) Timeline of the experiment, structural formula of (**B**) ginsenoside Rc and (**C**) imipramine, and (**D**) location of the L-AAA infusion. For injection of the prefrontal cortex, the following coordinates were used: AP 1.7 mm, ML ± 0.3 mm, and DV −2.5 mm from the bregma. Allen Mouse Brain Atlas, mouse.brain-map.org [108] (accessed on 8 July 2024) and atlas.brain-map.org [109] (accessed on 8 July 2024). L-AAA, L-alpha-aminoadipic acid; OFT, open-field test; FST, forced swimming test; TST, tail suspension test; AP, anterior–posterior; ML, medial–lateral; DV, dorsal–ventral.

All procedures were performed according to the schedule shown in Figure 5A. Experimental animals were randomly assigned to four groups: (i) sham control (sham), distilled water per os (po) + sham surgery (n = 9); (ii) L-AAA (100 μg/mL) + vehicle, distilled water po (n = 9); (iii) L-AAA + imipramine (IMI) (1 mg/mL) po (n = 9); and (iv) L-AAA + Rc (20 mg/kg) po (n = 9). Imipramine was selected as an active control in this study because it is a widely used and well-established antidepressant, allowing the effects of G-Rc to be compared with a standard treatment in clinical practice. G-Rc and IMI were completely dissolved in distilled water and orally administered to the mice. The dose of 20 mg/kg for G-Rc was selected based on studies of other ginsenosides on the nervous system, as there were no previous studies specifically investigating the effects of G-Rc on a mouse model of diseases in the central nervous system [20,103,104,105,106]. G-Rc (CFN99973; PubChem CID: 12855889; CAS Number: 11021-14-0; purity ≥ 98%) was purchased from ChemFaces, Inc. (Wuhan, China) (Figure 5B). G-Rc and IMI were prepared on a clean bench using sterilized distilled water, and all administration equipment, including oral gavage needles and syringes, was sterilized with ultraviolet light prior to use. Oral administration continued until the mice were euthanatized.

### 4.3. Cannula Implantation and Injection of L-AAA

This experiment was performed as previously described [25]. Mice were anesthetized using an intraperitoneal injection of Avertin at a dose of 240 mg/kg and placed on a stereotaxic apparatus (Vernier Stereotaxic Instrument; Leica Biosystems, Nussloch, Germany). A guide cannula (RWD Life Science Co., Ltd., Shenzhen, China) was implanted at the location shown in Figure 5D to inject the drug into the mouse’s PFC region.

After a week of recovery from implantation of the cannula, L-AAA was infused bilaterally at 0.1 μL/min for 6 min through an injection cannula using a micro-drive pump (Pump 11 Elite Nanomite; Harvard Apparatus, Holliston, MA, USA). L-AAA was administered once daily for two days. L-AAA was infused using a previously implanted guide cannula, except in the sham group.

### 4.4. Behavioral Tests

#### 4.4.1. Open-Field Test

The OFT was performed to assess locomotor activity and to evaluate potential sensorimotor deficits resulting from the intracerebral L-AAA infusion procedure. Each mouse was placed at the center of an acrylic box (50 × 50 × 50 cm) and allowed to freely explore the apparatus. The locomotor activities of the mice were recorded for 10 min using a video camera installed above the apparatus. The total distance traveled was analyzed using Smart 3.0 (Panlab Harvard Apparatus, Holliston, MA, USA).

#### 4.4.2. Tail Suspension Test

Acoustically and visually isolated mice were suspended 50 cm above the floor using adhesive tape. The immobility time was defined as the duration of passive and completely motionless hanging. The immobility time was analyzed during the last 4 min of the 6 min suspension period.

#### 4.4.3. Forced Swimming Test

Mice were forced to swim in an open cylinder (diameter, 20 cm; height, 35 cm) filled to a depth of 19 cm with water at 25 °C. The total time that the mice ceased to struggle and remained floating and motionless in the water was measured during the last 4 min of each 6 min testing period. Decreased immobility time was regarded as a result of the antidepressant effects.

### 4.5. Tissue Collection

PFC samples were excised from mice for ELISA. For immunofluorescence analysis, the mice were anesthetized, and anesthesia was assessed by testing the pedal reflex on their hind legs with forceps every 5 min to ensure proper status. The mice were perfused with phosphate-buffered saline, followed by 4% paraformaldehyde, using the transcardial perfusion method. After brain dissection, the brain tissue was fixed with 4% paraformaldehyde for 24 h at 4 °C and then sequentially submerged in 10%, 20%, and 30% sucrose solution for cryoprotection. Brain samples were stored at −80 °C.

### 4.6. ELISA

ELISA was performed using the following kits according to the manufacturer’s instructions: mouse LCN2/NGAL ELISA kits (MLCN20; R&D Systems, Minneapolis, MN, USA). The detection limit of the mouse LCN2/NGAL ELISA was 5000 pg/mL.

### 4.7. RNA Extraction and cDNA Synthesis

Total RNA was extracted from the brain tissue samples using TRIzol reagent. The quality and concentration of RNA were determined using a NanoDrop spectrophotometer (Thermo Fisher Scientific, Waltham, MA, USA). For cDNA synthesis, 1 µg of total RNA was reverse-transcribed into cDNA using the Thermo RevertAid First Strand cDNA Synthesis Kit (K1622, Thermo Fisher Scientific, Waltham, MA, USA), according to the manufacturer’s protocol.

### 4.8. Quantitative Real-Time PCR

Quantitative real-time PCR was performed using a StepOnePlus™ Real-Time PCR System (Thermo Fisher Scientific, Waltham, MA, USA) with Power SYBR™ Green PCR Master Mix (4368706, Thermo Fisher Scientific, Waltham, MA, USA). The cDNA was diluted 1:10 with nuclease-free water, and 2 µL of the diluted cDNA was used as a template in a 20 µL reaction volume. Each reaction contained 10 µL of Power SYBR™ Green Master Mix, 0.5 µL of forward primer (10 µM), 0.5 µL of reverse primer (10 µM), 2 µL of cDNA template, and 7 µL of nuclease-free water.

The thermal cycling conditions were as follows: initial denaturation at 95 °C for 10 m, followed by 40 cycles of 95 °C for 15 s and 60 °C for 1 min. A melting curve analysis was performed to verify the specificity of the amplification. The relative expression levels of target genes were normalized to the expression of the housekeeping gene GAPDH and analyzed using the 2^−ΔΔCt^ method. The sequences used were as follows: IL-6 (GenBank No: NM_031168): F, 5′-AGTTGCCTTCTTGGGACTGA-3′, R, 5′-TCCACGATTTCCCAGAGAAC-3′; TNF-α (GenBank No: NM_013693): F, 5′-ATGGCCTCCCTCTCAGTTC-3′, R, 5′-TTGGTGGTTTGCTACGACGTG-3′; TGF-β (GenBank No: NM_011577): F, 5′-CTCCCGTGGCTTCTAGTGC-3′, R, 5′-GCCTTAGTTTGGACAGGATCTG-3′; LCN2 (GenBank No: AA087193): F, 5′-ATGTCACCTCCATCCTGGTC-3′, R, 5′-CACACTCACCACCCATTCAG-3′; GAPDH (GenBank No: NM_008084): F, 5′-TGGGCTACACTGAGCACCAG-3′, R, 5′-GGGTGTCGCTGTTGAAGTCA-3′.

### 4.9. Immunofluorescence

The brain tissue was prepared as frozen sections using optimal cutting temperature (OCT) compound. The frozen sections were cut at a temperature of −20 °C into 10 μm thick slices using a cryostat (Leica CM1850, Leica Biosystems, Nussloch, Germany). Tissue slides were dried for 1 h before the experiment. Slides were immersed in 1× Tris-ethylenediaminetetraacetic acid buffer (pH 9.0) and warmed at 70–80 °C for 30 min for antigen retrieval. Slides were then washed with 1× Tris-buffered saline with Tween (TBST), and a circular line was drawn around the tissue using a liquid block pen. Tissues were treated with diluent (ADT999; ScyTek Laboratories, Logan, UT, USA) for 15 min (blocking procedure). Monoclonal mouse anti-mouse GFAP antibody (sc-33673) was purchased from Santa Cruz Biotechnology, Inc. (Heidelberg, Germany). Polyclonal rabbit anti-mouse IBA-1 antibody (PA5-27436) was purchased from Invitrogen (Carlsbad, CA, USA). Processed tissues were processed and incubated with diluted primary antibody “A” at 4 °C for 24 h, and then the tissues were washed with 1× TBST. The secondary antibody diluted in 1× TBST solution was incubated with tissue samples for 20 min in the dark. The secondary antibody was removed, and tissue samples were treated with primary antibody “B” and incubated at 4 °C for 24 h. Samples were then treated with diluted secondary antibodies, washed, and treated with DAPI (4083S, Cell Signaling Technology, Danvers, MA, USA) for 1 min, followed by a rinse. The samples were then mounted using FluoroGuard Mounting Medium (FMM060, ScyTek Laboratories, Logan, UT, USA), and cover slides were added. The co-localization of antibodies “A” and “B” was observed using a fluorescence microscope.

The microscope images were processed using ImageJ v.1.54a software (NIH, Bethesda, MD, USA). The number of GFAP- and IBA-1-positive cells per mm^2^ was manually counted in each image using ImageJ. To determine the average number of cells per mm^2^, the total cell counts for each region were divided by the total area. For measuring the mean area of GFAP- and IBA-1-positive cells, the images were converted to 8-bit grayscale. A threshold was applied to highlight the fluorescent areas. The area exceeding the threshold was calculated as a percentage.

### 4.10. Western Blot

Mouse brain tissue was homogenized in RIPA buffer (89900, Thermo Scientific™, Waltham, MA, USA) supplemented with protease and phosphatase inhibitors (1861281, Thermo Scientific™, Waltham, MA, USA). The homogenate was centrifuged (4 °C, 15 min, 12,000× *g*) to collect the supernatant. Protein concentration was determined using a BCA protein assay kit (23225, Thermo Scientific™, Waltham, MA, USA) and quantified with a Spark 10M microplate reader (Tecan, Männedorf, Switzerland). A total of 20 µg of protein extract was loaded onto a 12–15% SDS-PAGE gel and transferred to PVDF membranes (IPVH00010, Millipore, Billerica, MA, USA).

The primary antibodies used were GFAP (3670S, Cell Signaling Technology, Danvers, MA, USA), IBA-1 (PA5-27436, Invitrogen, Carlsbad, CA, USA), NeuN (ab177487, Abcam, Cambridge, UK), caspase-3 (9662, Cell Signaling Technology, Danvers, MA, USA), Bcl-2 (3498S, Cell Signaling Technology, Danvers, MA, USA), and β-Actin (4967, Cell Signaling Technology, Danvers, MA, USA). The primary antibodies were diluted 1:1000 in a 1% BSA solution. Membranes treated with the primary antibodies were incubated overnight at 4 °C with gentle shaking.

The secondary antibodies used were anti-rabbit (31460, Invitrogen, Carlsbad, CA, USA) for GFAP, IBA-1, caspase-3 and Bcl-2, and anti-mouse (31430, Invitrogen, Carlsbad, CA, USA) for NeuN and β-Actin. Protein bands were visualized using the Amersham Imager 600 (GE Healthcare, Chicago, IL, USA) for caspase-3, Bcl-2, and β-Actin, and the Davinch-chemi™ Chemiluminescence Imaging System (Davinch-K, Seoul, Republic of Korea) for GFAP, IBA-1, β-Actin, and NeuN, with ECL (34577, Thermo Scientific™, Waltham, MA, USA).

### 4.11. Statistical Analysis

Behavioral and biochemical analyses were performed using a one-way analysis of variance, followed by Tukey’s post hoc test. Results are expressed as the mean ± standard error of the mean; *p*-values < 0.05 were considered significant. SPSS version 22.0 (Armonk, NY, USA) was used for statistical analysis.

## 5. Conclusions

This study showed that G-Rc administration reduced depressive behavior in mice. Furthermore, inflammatory responses, astrocytic degeneration, and apoptosis in the PFC were decreased by G-Rc administration. These results were observed in animal models, similar to the brain features of patients with MDD, and G-Rc showed effects comparable to those of IMI. The multi-faceted action of G-Rc, addressing neuroinflammation, astrocyte function, and apoptotic pathways, suggests its potential as a novel antidepressant agent. Therefore, G-Rc, a component of *P. ginseng*, warrants further investigation as a promising therapeutic option for depression.

## Data Availability

Data supporting the findings of this study are available from the corresponding author upon request.

## Supplementary Materials

The following supporting information can be downloaded at: https://www.mdpi.com/article/10.3390/ijms25179673/s1.

## Abbreviations

ELISA	Enzyme-linked immunosorbent assay
FST	Forced swimming test
GABA	Gamma-aminobutyric acid
GFAP	Glial fibrillary acidic protein
G-Rc	Ginsenoside Rc
IBA-1	Ionized calcium-binding adapter molecule 1
IL-6	Interleukin-6
IMI	Imipramine
L-AAA	L-alpha-aminoadipic acid
LCN2	Lipocalin-2
MDD	Major depressive disorder
NeuN	Neuronal nuclei antigen
OCT	Optimal cutting temperature
OFT	Open-field test
PFC	Prefrontal cortex
Real-time PCR	Real-time polymerase chain reaction
TBST	Tris-buffered saline with Tween
TGF-β	Transforming growth factor-beta
TNF-α	Tumor necrosis factor-alpha
TST	Tail suspension test
