# Supplementary material for: Antidepressant Effects of Ginsenoside Rc on L-Alpha-Aminoadipic Acid-Induced Astrocytic Ablation and Neuroinflammation in Mice
Source: Int J Mol Sci. 2024 Sep 6;25(17):9673. doi: 10.3390/ijms25179673 (PMC11396248; doi:10.3390/ijms25179673)
Supplement: Supplementary file 1 [file ijms-25-09673-s001.zip › ijms-3122619-supplementary.pdf]

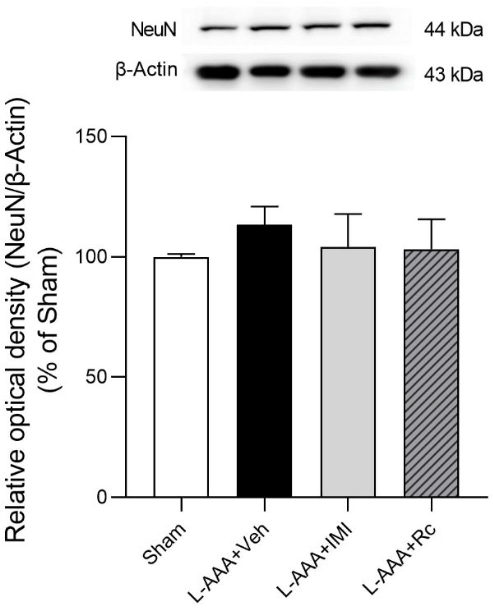

Supplementary Figure S1. Effects of G-Rc on NeuN following L-AAA administration. Western blot analysis of NeuN protein levels. Quantitative data are expressed as the mean ± SEM. N = 3 per group. G-Rc, ginsenoside Rc; L-AAA, L-alpha-aminoadipic acid; IMI, imipramine.
